# How familiar are our doctors towards Rabies prophylaxis- A study from coastal south India

**DOI:** 10.1371/journal.pntd.0006032

**Published:** 2017-10-30

**Authors:** Ramesh Holla, Bhagawan Darshan, Astha Guliani, Bhaskaran Unnikrishnan, Rekha Thapar, Prasanna Mithra, Nithin Kumar, Vaman Kulkarni, Avinash Kumar, Salman Anwar

**Affiliations:** 1 Department of Community Medicine, Kasturba Medical College (Manipal University), Mangalore, Karnataka State, India; 2 Undergraduate MBBS student, Kasturba Medical College (Manipal University), Mangalore, Karnataka State, India; Wistar Institute, UNITED STATES

## Abstract

**Background:**

Rabies, a 100% fatal disease claims more than 59,000 human lives every year globally. One human life is lost every 15 minutes due to this deadly preventable disease. Timely initiation of post exposure prophylaxis following an animal exposure can result in 100% preventability of this fatal disease.

**Methodology:**

This facility based study was conducted among clinical fraternities of teaching hospitals. A semi structured questionnaire was used for collection of data. Institutional Ethics Committee approval was sought. The study investigators visited the workplace of the participants and distributed the questionnaire. SPSS Ver 16 (Chicago, IL, USA) was used to analyse the data.

**Findings:**

Most of the participants knew that veterinary groups and zoo-keepers should be given pre-exposure prophylaxis. Many participants knew about the Intra Muscular schedule of anti-rabies vaccine and its site of administration for pre exposure prophylaxis. It was observed that most participants had knowledge regarding correct intramuscular regimen of anti-rabies vaccine for post-exposure prophylaxis but less than half were able to differentiate between the intramuscular and intradermal regimens. Less than half of participants were aware of the fact that local administration of anti-rabies serum is useful.

**Conclusion:**

The knowledge regarding WHO categorisation of animal exposure and recommended post exposure prophylaxis according to type of exposure observed to be minimal among clinical fraternity.

## Author summary

Rabies is a zoonotic disease. More than 95% of human rabies deaths is dog mediated. Rabies a 100% fatal yet preventable disease; a major public health problem in most of the Asian countries including India. The reduction in human rabies deaths is possible only when our clinical fraternity are aware of WHO recommended rabies post exposure prophylaxis to be offered to animal bite victims. With this in mind, a study was done among clinical fraternities and post graduates of Medicine, Surgery and Paediatrics departments of three tertiary care teaching hospitals affiliated to a medical college. It was observed that, the knowledge regarding reservoir of rabies infection and modes of transmission of rabies infection was satisfactory but the knowledge regarding WHO categorisation of animal exposure and recommended rabies post exposure prophylaxis was inadequate. Bridging this knowledge gap through periodically conducted Continuous Medical Education, Workshops and hands on training is essential for reducing human rabies deaths.

## Introduction

Rabies, a neglected tropical zoonotic disease is caused by the rabies virus (Lyssavirus genus, family Rhabdoviridae). Globally 95% human rabies’ mortality can be attributed to rabid dog exposure [1]. Majority of death toll due to human rabies predominantly occurs among poor rural community particularly amongst children, where the accessibility and affordability to the rabies immunologicalsis still a mirage [1].

Rabies, a 100% fatal disease claims more than 59,000 human lives every year globally. One human life is lost every 15 minutes due to this deadly preventable disease [2]. Asian countries contribute nearly half of rabies deaths. In India, rabies still poses a major public health threat. An Indian multi-centric study conducted in the year 2003 estimates that around 20,000 human rabies deaths occur annually [3].

Timely initiation of post exposure prophylaxis following an animal exposure can result in 100% preventability of this fatal disease [3]. Studies conducted in various parts of the globe have revealed a low level of awareness among the health care professionals in rabies post exposure prophylaxis [4–9]. Further reduction in the incidence of human rabies is possible only when WHO recommended rabies post exposure prophylaxis is provided to animal bite victims by the clinical fraternity who are the first line of contact following an animal exposure. So, the present study was designed to assess the existing knowledge amongst post-graduates and clinical faculty, regarding prophylaxis of rabies in the district hospital which also acts as a referral centre for other districts of coastal Karnataka and northern part of Kerala.

## Materials and methods

### Study design and participants

The present facility based study was conducted in 2014 at three different tertiary care teaching hospitals (two government and one private health care facility) affiliated to our medical college situated in Mangalore. This study was done among clinical fraternities and post graduates of Medicine, Surgery and Paediatrics departments. The sample size of 96 was obtained based on a study; where 81.4% [10] of the clinical fraternities were aware of wound management and post exposure prophylaxis for Rabies. Confidence level was kept at 95% and an absolute precision of 10% was considered while determining the sample size. The number of clinical fraternities and postgraduates was decided based on probability proportion to size technique.

### Study instrument

A semi structured questionnaire was formulated after extensive literature search and by consulting experts in the field of Rabies. Due modifications were done to the questionnaire based on the feedback obtained through pilot study. The final questionnaire consisted of 4 sections. The first section included details related to personal characteristics of study participants. Awareness regarding epidemiological determinants of Rabies was included in second section. Section 3 and Section 4 included the questionnaire related to Rabies Post exposure prophylaxis and pre-exposure prophylaxis respectively. To increase the precision of the responses and freedom from busy clinical schedule, it was coordinated with each clinical faculty member to provide a questionnaire during the time when the outpatient attendance was minimal.

### Ethics statement

The principle of ethics in research was followed in the present study for questioning and collection of data. The research proposal was presented before the Institutional Ethics Committee and obtained approval from Institutional Ethics Committee. Permission was also obtained from the Medical Superintendents of the hospitals affiliated to Medical College. The study investigators visited the workplace of the participants and distributed the questionnaire to fill out. The written informed consent to participate in the study was obtained from the study participants after fully explaining the study purpose and ensuring them of the confidentiality of their data.

### Statistical analysis

SPSS Ver 16 (Chicago, IL, USA) was used to analyse the data. Results were expressed in proportions, mean and standard deviation.

## Results

The mean age of the study participants was 31.46 (±8.478) years and ranged from 24 to 50 years. Majority of the study participants were less than 30 years of age with males being predominant. Maximum participants were from Medicine Department, followed by Surgery and Paediatrics. Most of the participants had less than 5 years of clinical experience. (Table 1)

**Table 1:** Baseline characteristics of the study participants (n = 96).

BASELINE CHARACTERISTICS	Number	PERCENTAGE
**Age Group (years)**		
≤30	66	68.8
31–40	13	13.5
>40	17	17.7
**Gender**		
Male	67	69.8
Female	29	30.2
**Department**		
Medicine	44	45.8
Surgery	35	36.5
Paediatrics	17	17.7
**Designation**		
Professor	08	08.3
Associate Professor	07	07.3
Assistant Professor	24	25.0
Post-Graduates	57	59.4
**Years of experience (years)**		
≤5	71	74.0
6–15	13	13.5
>15	12	12.5

Among the participants who answered the question, majority were aware that rabies is not transmitted by licks of rabid animals over intact skin while very few knew that licks over broken skin comes under Category III. A good number of participants were aware that saliva is the reservoir for rabies infection. More than half thought that even Bats were responsible for the spread of rabies in India and many were aware that body fluids of rabies patients are infectious. About half of the participants knew that usual incubation period of rabies in humans is 1–3 months. (Table 2)

**Table 2:** Distribution of study participants according to their knowledge regarding epidemiological determinants of rabies.

STATEMENT	CORRECT RESPONSE	PERCENTAGE OF CORRECT RESPONSES
Rabies is not transmitted by: (n = 92)	Licks of rabid animal over intact skin	91.5
Reservoir for rabies infection: (n = 96)	Saliva	93.8
In India, rabies is not transmitted by: (n = 92)	Bats	45.7
Is body fluid of rabies patient infectious? (n = 96)	Yes	61.5
Usual incubation period of rabies in humans: (n = 94)	1–3 months	50.0
Licks over broken skin is categorised as: (n = 95)	Category III	13.6
Days of observing dog after bite: (n = 96)	10–14 days	89.6
Chances of survival after 1^st^ clinical symptoms: (n = 95)	<1%	66.3

Most of the participants knew that veterinary groups and zoo-keepers should be given pre-exposure prophylaxis. Many participants knew about the Intra Muscular schedule of anti-rabies vaccine and its site of administration. About half of the participants knew that infant and adult dose of anti-rabies vaccine is the same. (Table 3)

**Table 3:** Distribution of study participants according to their awareness regarding pre-exposure prophylaxis.

STATEMENT	CORRECT RESPONSE	PERCENTAGE OF CORRECT RESPONSES
Groups to be given pre-exposure prophylaxis: (n = 95)	Both veterinary doctors & zoo-keepers	92.6
Intra Muscular schedule of anti-rabies vaccine: (n = 94)	Days 0, 7 and 28	88.3
Site of administration of Intra Muscular vaccine: (n = 96)	Deltoid	70.8
Dose of anti-rabies vaccine in infants: (n = 95)	Same as adult dose	55.8

92.7% of participants were aware that suturing of bite-wound is not to be done while only 27.1% knew that the site of administration of RIG is into and around the wound. More than half of the participants knew that dressing of bite-wound wasn’t necessary, bite wound should not be cauterized, local administration of anti-rabies serum is useful, rabies vaccine is non-neural, 5 Intra Muscular doses are given for rabies PEP and patient has to be started with full PEP if he/she comes 1 week after the bite. Less than half of the participants knew the minimum duration of washing bite wound with water, difference between Intra Muscular and Intra Dermal regimen and vaccination schedule of re-exposure after 6 months of complete PEP. (Table 4)

**Table 4:** Distribution of study participants according to their awareness regarding post-exposure prophylaxis (PEP).

STATEMENT	CORRECT RESPONSE	PERCENTAGE OF CORRECT RESPONSES
Is dressing of bite-wound necessary? (n = 96)	No	70.8
Minimum duration of washing bite-wound with water: (n = 95)	10–15 mins	46.3
Is suturing of bite-wound necessary? (n = 96)	No	92.7
Can bite-wound be cauterized? (n = 96)	No	75.0
Type of rabies vaccine for PEP: (n = 95)	Non-neural	73.7
Intra Muscular regimen for rabies PEP: (n = 96)	5 doses	83.3
Difference between Intra Muscular and Intra Dermal regimen: (n = 88)	No vaccine on day 14	40.9
If orange-peel appearance is absent: (n = 88)	Repeat the dose	46.6
Is local administration of anti-rabies serum useful? (n = 96)	Yes	69.8
Site of administration of Rabies immunoglobulin (RIG): (n = 96)	Full dose into & around the wound	27.1
Respective doses of Equine RIG & Human RIG (IU/kg): (n = 92)	40 / 20	69.6
If skin-sensitivity is (+)ve for ERIG: (n = 94)	Change over to HRIG	85.1
If amount of RIG is inadequate to infiltrate all wounds: (n = 91)	Dilute with normal saline & infiltrate all wounds	54.9
If patient comes 1 week after bite: (n = 95)	Start with full PEP	82.1
If patient comes with re-exposure after 6 months of complete PEP: (n = 93)	Give vaccine on days 0 & 3	39.8

## Discussion

Measures to control rabies effectively must be taken considering the burden of rabies in India. Public education campaigns need to be conducted to make people aware of the existence of rabies, especially in remote areas, and for spreading awareness about the vital importance of seeking medical care immediately after an animal bite. But before that, the existing knowledge amongst post-graduates and clinical faculty regarding the animal bite wound management and rabies prophylaxis must be assessed.

In our study, the mean age of the study participants was 31.46 (±8.48) years and they were predominantly males. The studies done in Turkey had a similar age distribution [9, 11]. The studies carried out in Northern part of India [10, 12] had a higher mean age group while the study in Kolkata had a lower mean age [4]. The mean work experience was found to be lower in the studies conducted in Istanbul [9] and Kolkata [4], while it was higher in other studies [10, 11, 12].

Majority of the participants were aware that rabies is not transmitted by licks of rabid animals over intact skin while very few knew that licks over broken skin can be grouped under Category III exposure. In another study conducted in Ambala, on being asked if rabies could be transmitted by routes other than dog bite, 31% answered that it can be transmitted by blood transfusion, faeco-oral, sputum, or via the perinatal route [10]. In a study conducted in France, more than 80% participants knew which animals transmitted the disease as well as the severity criteria for bites [13]. However in a study done at Istanbul, less than half of participants opined that mucosal contact may also lead to rabies transmission [9]. While in Sanliurfa, 96.4% of the physicians correctly indicated that cats and dogs can transmit the disease, the fact that foxes also have a role in transmission was known only by 48.8% [11]. The knowledge regarding the incubation period of the disease was 54% which is consistent with studies done across the world [9–11,14]. More than four fifth of physicians were able to answer the correct duration of observation of the rabid animal which is consistent with the study done in Ambala and was much higher than the one conducted in Istanbul [9,10]. In contrast to the findings of Ambala and Istanbul, correct knowledge of categorization of animal bite was lower in findings of present study and Belgaum [9,10,15]. A striking gap was noted between the present study and studies done in central part of India and Turkey regarding pre-exposure prophylaxis schedule [10,11].

In this study, the minimum duration for washing the bite wounds was known by less than half of the participants which was consistent with the study done at Ambala, whereas it was only one third in a study conducted in Istanbul [9, 10]. None of the participants in the Pakistan study knew that washing the wound with soap and water was useful [14]. The fact that knowledge regarding bite wound should not be dressed was observed among less than three fourth of study participants, whereas it was 40% and 94% in studies conducted at Belgaum and Ambala [10, 15]. In line with the studies done at Istanbul and Kolkata, higher proportion of the study participants knew animal bite wound should not be sutured [4, 9].

In congruence with the findings of the present study nearly three fourth of the study participants from Istanbul were aware that non-neural vaccines are used for post-exposure prophylaxis [4]. In contrast, only 1.4% had knowledge regarding the same in Pakistan [14]. This could be because nerve tissue vaccines are being still used in Pakistan [14]. It was evident from the findings of our study that most participants had knowledge regarding correct intramuscular regimen of anti-rabies vaccine for post-exposure prophylaxis but less than half were able to differentiate between the intramuscular and intradermal regimens. Variance in the knowledge regarding the same was evident in different studies conducted across India [12, 15, 16]. Most participants of our study and the Ambala study were aware of the usefulness of local administration of rabies immunoglobulin in post-exposure prophylaxis [10]. Knowledge regarding the same was found to be minimal in studies done elsewhere [12, 14]. In all the studies that have been reviewed including ours, the knowledge regarding the site of administration of immunoglobulin among the participants was low [4, 9, 10].

It was positive to note that more number of participants were aware of the dosage of ERIG and HRIG as compared to other studies [4,9]. Proportion of knowledge regarding vaccination schedule to be followed in a case of re-exposure after being completed with PEP in the past was in congruence with the study findings conducted in other parts of India and globe [9, 11].

In conclusion, the knowledge regarding WHO categorisation of animal exposure and recommended post exposure prophylaxis according to type of exposure observed to be inadequate among clinical fraternity of medical and surgical branches across all the cadres. Successful implementation of rabies elimination strategy is possible only when there is capacity building of clinical fraternity on WHO recommended rabies prophylaxis through periodically conducted Continuous Medical education, Workshops and hands on training.

## Supporting information

S1 Dataset (SAV)
